# Global field synchronization reveals rapid eye movement sleep as most synchronized brain state in the human EEG

**DOI:** 10.1098/rsos.160201

**Published:** 2016-10-12

**Authors:** Peter Achermann, Thomas Rusterholz, Roland Dürr, Thomas König, Leila Tarokh

**Affiliations:** 1 Institute of Pharmacology and Toxicology, University of Zurich, Zurich, Switzerland; 2 Zurich Center for Integrative Human Physiology, University of Zurich, Zurich, Switzerland; 3 Zurich Center for Interdisciplinary Sleep Research, University of Zurich, Zurich, Switzerland; 4 Neuroscience Center Zurich, University of Zurich and ETH Zurich, Zurich, Switzerland; 5 University Hospital of Child and Adolescent Psychiatry and Psychotherapy, University Hospital of Psychiatry, University of Bern, Bern, Switzerland; 6 Translational Research Center, University Hospital of Psychiatry, University of Bern, Bern, Switzerland; 7 Department of Psychiatry and Human Behavior, The Alpert Medical School of Brown University, Providence, RI, USA

**Keywords:** sleep, EEG, global field synchrony, rapid eye movement sleep

## Abstract

Sleep is characterized by a loss of consciousness, which has been attributed to a breakdown of functional connectivity between brain regions. Global field synchronization (GFS) can estimate functional connectivity of brain processes. GFS is a frequency-dependent measure of global synchronicity of multi-channel EEG data. Our aim was to explore and extend the hypothesis of disconnection during sleep by comparing GFS spectra of different vigilance states. The analysis was performed on eight healthy adult male subjects. EEG was recorded during a baseline night, a recovery night after 40 h of sustained wakefulness and at 3 h intervals during the 40 h of wakefulness. Compared to non-rapid eye movement (NREM) sleep, REM sleep showed larger GFS values in all frequencies except in the spindle and theta bands, where NREM sleep showed a peak in GFS. Sleep deprivation did not affect GFS spectra in REM and NREM sleep. Waking GFS values were lower compared with REM and NREM sleep except for the alpha band. Waking alpha GFS decreased following sleep deprivation in the eyes closed condition only. Our surprising finding of higher synchrony during REM sleep challenges the view of REM sleep as a desynchronized brain state and may provide insight into the function of REM sleep.

## 1. Introduction

Sleep is a behaviour observed in all organisms studied thus far. Among the behavioural definition of sleep is a significant decline in responsiveness to external stimuli. This reduction in responsiveness, or consciousness, during sleep has been attributed to the breakdown of connectivity between brain regions [1]. According to this idea, called the integrated information theory, consciousness arises when information is integrated across brain regions [1]. Support for this conjecture comes from experimental studies that have used simultaneous high-density EEG and transcranial magnetic stimulation (TMS) to examine signal propagation during waking and sleep. These studies have found that when compared with waking, in which the TMS-induced signal propagates across brain regions for up to 300 ms, during early non-rapid eye movement (NREM) sleep the activity induced by TMS remains localized to the site of stimulation and lasts less than 150 ms [2]. As compared to early NREM sleep, the TMS-evoked response during REM sleep, a state often associated with vivid dreams, was more widespread and longer in duration [3]. However, the TMS response during REM sleep was more localized than that observed during waking, suggesting that the response to REM sleep is in between that of waking and NREM sleep.

Although TMS is a useful tool to probe brain connectivity, it is not a measure of spontaneous cortical activity. One of many ways to measure spontaneous connectivity during different states is through calculating EEG coherence. Simply put, coherence is a measure of the degree of correlation between two EEG derivations in the frequency domain. Large coherence values are believed to indicate functional connectivity between two regions or indicate that a common third region drives both regions [4]. Studies of coherence during sleep have indicated that coherence is higher during NREM when compared with REM sleep in the delta and sigma bands [5,6].

Global field synchronization (GFS) is a method to measure large-scale synchrony introduced by Koenig *et al.* [7]. This measure uses multi-channel EEG to get a measure of global phase alignment across all derivations as a function of frequency. GFS measures phase synchronization between all derivations and ranges from zero (no predominant phase; minimal phase synchronization among derivations) to one (perfect phase synchronization; all derivations in phase or anti-phase; [7]). Given that the EEG signal is not produced by a single, focal electric source, the observation of a predominant phase across the scalp measurements implies that there is also a preferred phase for the intracranial neuroelectric dynamics, and a spread in phase across scalp measurements must have been generated by intracranial electric sources that differ in phase. This measure has successfully been used to show differences in functional connectivity in schizophrenic and Alzheimer patients as compared to controls [7–9]. Furthermore, applying this method to the anaesthetized brain revealed decreased global synchrony in the gamma range (30.5–80 Hz) [10].

Given that GFS has been shown to be a measure of large-scale synchrony, we apply this method to the sleep EEG of healthy adults during baseline sleep after 16 h of wakefulness in order to compare synchrony in waking, and two sleep states—NREM and REM sleep. Furthermore, we examine GFS following 40 h of sustained wakefulness to determine whether sleep deprivation has an impact on global synchrony. Such sleep deprivation induces stereotypical changes to the sleep EEG power spectrum, most prominently an increase in delta power (0.75–4.5 Hz) [11]. In addition to the effects of sleep deprivation on the sleep EEG, waking EEG theta power (5–8 Hz) increases with time awake [12]. Thus, we also examined GFS of the waking EEG as a function of time awake.

## 2. Material and methods

### 2.1. Dataset

The analyses were performed on an existing dataset of eight healthy young male participants of a previous study investigating the effects of sleep deprivation on EEG topography [12,13]. Polysomnographic recordings were obtained during an adaptation night, a subsequent baseline night and a recovery night after 40 h of sustained wakefulness. Waking EEG recordings, consisting of 5 min eyes open, 4–5 min eyes closed and a second 5 min eyes open session, were performed every 3 h during the 40 h sleep deprivation period which started at 07.00 h. During baseline and recovery sleep and for the waking EEG, 27 scalp EEG electrodes (extended 10–20 system; locations shown in the electronic supplementary material, figure S1) were recorded. Bedtime for all three nights was scheduled at 23.00 h and sleep was limited to 8 h for the adaptation and baseline nights and to 12 h for the recovery nights. Participants were instructed to abstain from alcohol and to adhere to regular bedtimes (8 h time in bed) for 3 days prior to the study, verified by ambulatory activity monitoring and sleep–wake diaries. The study protocol and all experimental procedures were approved by the local ethics committees for research on human subjects and participants gave their written informed consent. Sleep EEG data of baseline and recovery sleep after 40 h of sustained wakefulness were analysed. Waking EEG data at 3 h intervals was also analysed. The EEG signals were sampled at 128 Hz during sleep and at 256 Hz during wakefulness (for additional details, see [12,13]).

### 2.2. Data analysis

Sleep stages were visually scored for 20 s epochs (C3A2 derivation) according to the criteria of Rechtschaffen & Kales [14]. The analysis was restricted to the maximal common length of 7 h 32 min after sleep onset. However, in one subject 4 h 7 min of data were included due to technical problems (see [13] for details). Artefacts were identified as described by Finelli *et al.* [12,13].

### 2.3. Global field synchronization

GFS was introduced by Koenig *et al.* [7] and is briefly summarized here. The EEG was re-referenced to average reference. For consecutive 4 s epochs global functional connectivity was computed in the following way: using the fast Fourier transform with a Tukey window (tapered cosine, ratio of cosine-tapered section length to the entire window length = 0.2), the complex spectrum was determined for each derivation yielding a complex value for each frequency and derivation. At a given frequency, the complex Fourier coefficients of every channel can be mapped onto the complex plane (electronic supplementary material, figure S2). The shape of the resulting cloud of points is indicative of the amount of phase synchronization across derivations: a very elongated cloud indicates that the EEG at the given frequency is dominated by a common phase or anti-phase across all derivations. By contrast, if the cloud is nearly round, no predominant phase is present. To quantify the shape of the cloud in the complex plane, a two-dimensional principal component analysis (PCA) was applied by fitting an elliptical formed bivariate Gaussian distribution where the ratio of the principal axes correspond to the ratio of the PCA eigenvalues (*λ*_1_ and *λ*_2_). The ratio of the resulting two eigenvalues defines GFS:

2.1 $$\text{GFS( }f\text{) }=\frac{\left|\lambda_{1}(\;f)-\lambda_{2}(\;f)\right|}{\lambda_{1}(\;f)+\lambda_{2}(\;f)}.$$;

GFS ranges from 0 (no predominant phase; minimal synchronization) to 1 (all derivations in phase or anti-phase; maximal synchronization; [7]) and is independent of the spectral power. We note that it is crucial to apply an appropriate window function (non-rectangular) for determining GFS to avoid spurious synchronization in the beta/gamma range.

GFS makes no assumption about the spatial location of the activity and is independent of the reference [15]. The concept of GFS assumes that most types of measurable brain activity generate signals that are distributed over many electrodes on the scalp. Theoretically, an increase in GFS can have two possible interpretations. On the one hand, it may result from the increased activity of a single source which would be paralleled by an increase in spectral power. On the other hand, increased GFS can result from several active sources that increase their relative amounts of activity operating in a common phase such that they appear as a single meta-generator. The latter scenario refers to the classic binding hypothesis and does not necessarily imply an increase in spectral power [16].

For statistical analysis, GFS was Fisher *z*-transformed and back transformed for illustration. We analysed the entire GFS spectra during extended waking, baseline sleep and recovery sleep. We performed bootstrap analyses at each frequency bin (0.25 Hz frequency resolution; 0.5–25 Hz range) in order to test whether there were differences in GFS spectra between two states. Although statistical analysis was performed for all bins, we note that a 9-point moving average was used to smooth the spectra for clearer visualization in the figures. The bootstrap test is a non-parametric test and is particularly well suited to spectral analysis as it accounts for multiple comparisons [17]. We made the following comparisons using the bootstrap statistic: (i) in order to confirm that sleep deprivation has an impact on *power* spectra in the current dataset, we compared NREM and REM spectra before and after sleep deprivation, (ii) in order to examine the impact of sleep deprivation on GFS spectra, GFS spectra before and after sleep deprivation for NREM and REM sleep (separately) were compared, (iii) NREM and REM sleep GFS spectra in the baseline (no sleep deprivation) condition were compared to examine *sleep* state differences in GFS spectra, and (iv) waking GFS spectra after 15 h awake and NREM and REM sleep GFS spectra separately were compared to examine state differences in GFS spectra. The waking GFS spectrum after 15 h awake was chosen because it was the recording closest to when sleep would occur under normal, non-sleep-deprived circumstances.

In order to test the evolution of GFS in the theta (5–8 Hz) and alpha (9–11 Hz) bands across the sleep deprivation period, we performed a mixed models ANOVA with between-subjects factors time awake (14 time points) and eyes open/closed.

## 3. Results

### 3.1. State dependence of global field synchronization

**Figure 1.**
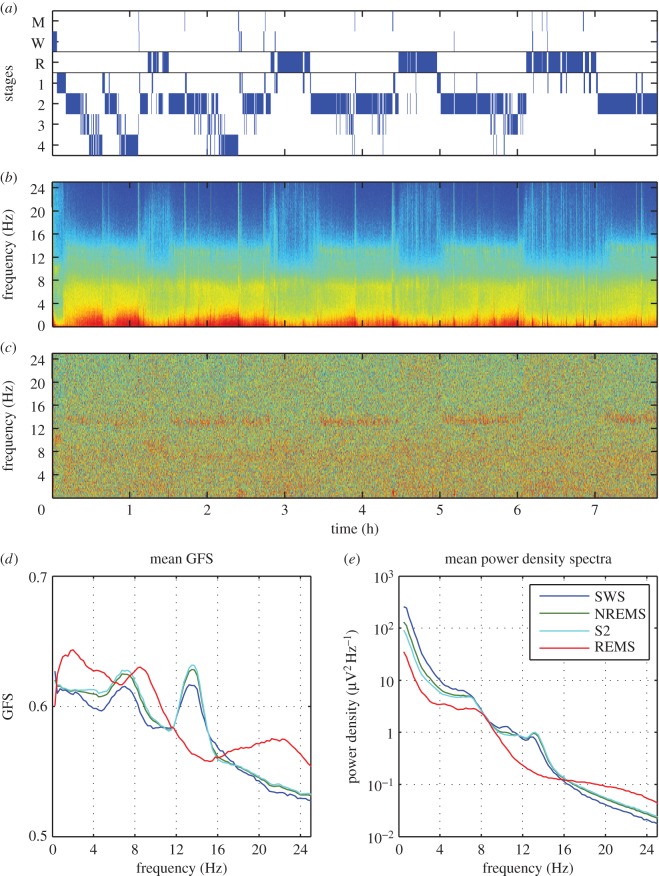
GFS and spectral power of a baseline night in one subject. (*a*) Hypnogram (M, movement time; W, waking; R, REM sleep; 1–4, stages of NREM sleep). (*b*) Spectrogram (colour-coded power density spectra of 20 s epochs) of the sleep EEG. (*c*) Colour-coded GFS spectra (4 s epochs). (*d*) Across the night average GFS spectra (smoothed, 9-point moving average) of slow wave sleep (SWS; stages 3 and 4), NREM sleep, stage 2 (S2) and REM sleep. (*e*) All night average power density spectra of SWS, NREM sleep, S2 and REM sleep. Note: all power density spectra are mean values of 27 derivations (average reference).

**Figure 2.**
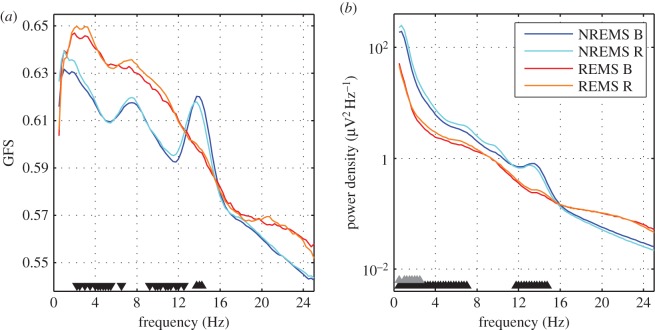
Subject average GFS spectra (smoothed, 9-point moving average; only for visualization purposes; (*a*)) and power density spectra (average across all 27 derivations; (*b*)) of NREM and REM sleep EEG during baseline (B) and recovery (R) sleep after total sleep deprivation (40 h of sustained wakefulness). Black triangles depict frequency bins that were significantly different between NREM and REM sleep for baseline recordings, grey triangles depict frequency bins that differed between recovery and baseline in NREM sleep (*p *< 0.05 derived from bootstrap analysis). The orientation of the triangles indicates the direction of the difference. GFS spectra of NREM and REM sleep EEG and power density spectra of the REM sleep EEG did not differ between baseline and recovery sleep.

The results of one representative subject for the baseline night of sleep are shown in figure 1. In this figure, sleep stages are shown in figure 1*a*, the power density and GFS spectrograms across the night as a function of frequency in figure 1*b* and figure 1*c*, respectively. In contrast to the power spectrogram, the GFS spectrogram does not show a clear modulation by the NREM-REM sleep cycle. Similar to the power density spectrogram, however, a band is visible in the spindle frequency range (i.e. between 12 and 16 Hz). The increase in GFS in the spindle frequency during NREM sleep is also reflected in the all-night mean of stage 2 (S2), slow wave and NREM sleep depicted in figure 1*d*. Surprisingly compared to slow wave sleep (SWS), S2 and NREM sleep, GFS was higher in most frequency bands during REM sleep. Two exceptions to this were the two peaks in the GFS spectrum—the spindle band in which GFS was higher in NREM sleep and the theta band in which the NREM and REM spectra did not differ. This is in contrast to the power density spectrum where power in NREM, S2 and SWS is above that of REM sleep up to 16 Hz. This trend was apparent in the single subject (figure 1) data and also in the mean (figure 2).

### 3.2. Effects of sleep deprivation on global field synchronization and power density spectra during sleep

Consistent with the published literature, we found an increase in spectral power during NREM sleep in the delta band and no change for REM sleep [11]. We note that the range is narrower than previously reported due to the use of the bootstrap statistic, which is more conservative and corrects for multiple comparisons. However, we found no impact of sleep deprivation on GFS during NREM or REM sleep (figure 2). Thus, GFS was highest during baseline/recovery REM sleep when compared with baseline/recovery NREM sleep (figure 2; with the exception of the spindle and theta band).

### 3.3. Effects of sleep deprivation on global field synchronization during sustained wakefulness

**Figure 3.**
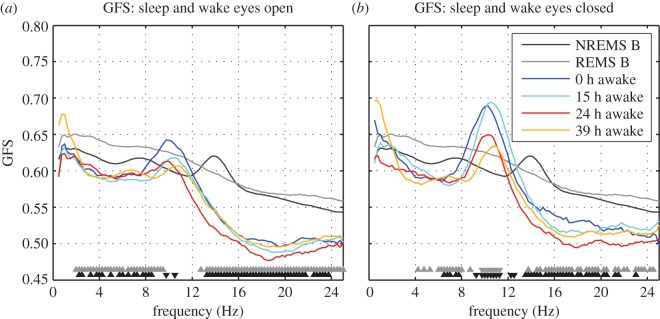
Subject average GFS spectra (smoothed, 9-point moving average; only for visualization purposes) of NREM, REM sleep and four different ‘doses’ of wakefulness. Eyes open waking GFS spectra shown in (*a*) and eyes closed in (*b*). Bootstrap statistics were used to compare NREM (significant bins shown in black triangles) and REM sleep (significant bins shown in grey triangles) with 15 h of wakefulness. The direction of the triangle shows the direction of the effect—up: sleep > waking and down: waking > sleep.

Interestingly, GFS was higher during sleep (NREM and REM sleep) when compared with waking in the eyes open and closed conditions (figure 3). An exception was GFS in the alpha band, which was greater in waking than NREM and REM sleep in the eyes closed condition (9–11 Hz). With regards to the eyes open condition, GFS was not significantly different between the two conditions in this band, with the exception of a few frequency bins. Furthermore, in both eyes open and closed conditions, there were no differences between waking and sleep in the low delta band (less than 2 Hz).

**Figure 4.**
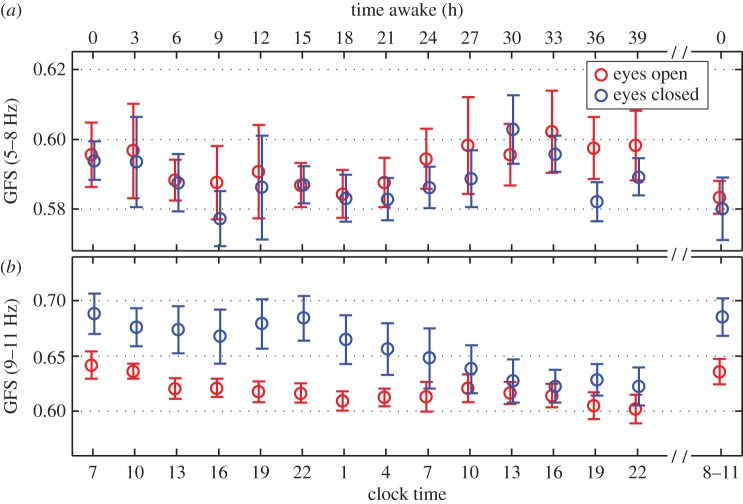
Time course of GFS in the theta (5–8 Hz) and alpha (9–11 Hz) band of the wake EEG during sustained wakefulness of 40 h. Time awake (*a*) and clock time (*b*) are indicated. The last data points are after a night of recovery sleep following sleep deprivation. GFS decreased as a function of sleep deprivation in the eyes closed condition only. We note that one subject did not contribute to the 9 h after waking (clock time = 16 h) or the 24 h after waking (clock time = 7 h).

In order to assess the impact of increasing time awake on waking EEG GFS, we chose two bands that appeared to be most impacted by time awake based on visual inspection (electronic supplementary material, figure S3)—the theta (5–8 Hz) and alpha (9–11 Hz) bands. Furthermore, previous studies have shown that these bands are sensitive to prolonged periods of waking [12,18,19]. Subject average and standard deviation of GFS in these bands as a function of time awake and clock time is shown in figure 4 for eyes open (red circles) and closed (blue circles). In the theta band no impact of increasing time awake was found for either eyes open or closed (main effect time, *p* = 0.26). Furthermore, there was a trend towards a significant effect for eyes open/closed conditions in this band (main effect condition, *p* = 0.054) and no significant interaction (*p* = 0.98).

On the other hand, GFS in the alpha band showed a main effect of time (*p* < 0.001) and condition (*p* < 0.001), with a trend towards an interaction (*p* = 0.058). Based on these results, we used a paired *t*-test to examine the effect of sleep deprivation on GFS in the alpha band. For this analysis, we averaged GFS prior to sleep deprivation to the corresponding time points following a night of sleep deprivation (10.00–22.00). We found a significant decline in GFS following sleep deprivation in the eyes closed condition (*p* = 0.02) but no change in the eyes open condition (*p* = 0.15).

## 4. Discussion

In this study, we examined GFS during three different states—waking, NREM and REM sleep—and found strong state-dependent modulations of GFS. In contrast to what is observed in power spectral measures, we find greatest global synchrony (GFS) during REM sleep when compared with NREM sleep and waking. This disassociation between power and GFS is not surprising given that GFS is independent of power and is a measure of phase synchrony across all derivations. Thus, such synchronous activity is not detectable by visual inspection and is different from EEG coherence as coherence is always between a pair of derivations. Therefore, our analysis of GFS reveals unique information about global synchrony across the brain.

### 4.1. Effects of sleep deprivation on global field synchronization during sleep

Sleep EEG NREM delta power is a reliable measure of sleep homeostasis—increasing with prolonged wakefulness and dissipating over the course of sleep. Unlike sleep EEG delta power, we see no impact of sleep deprivation on GFS in any frequency band for NREM or REM sleep. Furthermore, we do not find a decrease in GFS across the course of a night (figure 1), suggesting that sleep pressure does not impact brain synchrony. This finding is similar to what is seen in measures of coherence, which also do not change across a night of sleep [5]. This suggests that increased sleep depth does not result in a breakdown or strengthening of global synchrony.

### 4.2. Effects of sleep deprivation on global field synchronization during sustained wakefulness

Previous analysis of this dataset using power measures revealed a circadian modulation of theta power combined with an increase in power in this band with increasing time awake [12]. On the other hand, in contrast to power, we did not observe a change in GFS with increasing sleep pressure in the theta band, but rather in the alpha band. Others have reported a sensitivity of waking alpha power to increased time awake [18,19]. Meisel *et al.* [20] observed an increase of synchrony in a broad frequency range during sustained wakefulness (eyes open), whereas we found no change in GFS during the eyes open condition. This observation of increased synchrony was interpreted as increased cortical excitability as a function of time awake. However, the applied synchronization measure was based on phase synchrony calculated for all pairs of derivations and then averaged [20]. Since the measure used by Meisel and colleagues is an average, the increased synchronization may be driven by a subset of derivations and does not reflect global synchronization such as it is captured with GFS. Thus, by calculating GFS we uncover unique information about global synchrony during extended wakefulness.

### 4.3. Thalamocortical synchrony underlying global field synchronization?

We observed highest synchronization for the spindle band during NREM sleep, the alpha band during waking and REM sleep in general. It has long been established that NREM sleep spindles are generated through thalamocortical loops (e.g. [21]). Furthermore, neuroimaging studies in humans combining EEG with positron emission tomography (PET) or magnetic resonance imaging (MRI) have also implicated the thalamus in the generation of the waking alpha rhythm, with greater thalamic activity being associated with greater waking alpha power [22–25]. Finally, imaging studies of sleep have revealed high activity of the thalamus during REM sleep [26,27], and one study has reported widespread thalamocortical synchronized activity during phasic REM sleep [28]. Given that the thalamus plays an important role in the rhythms with the highest GFS—namely NREM sleep spindles, waking alpha and REM sleep in general—we hypothesize that the thalamus may be responsible for widespread cortical synchrony. Indeed, the thalamus projects widely to the cortex and is capable of synchronizing activity across cortical networks [29,30]. As such, the thalamus is an ideal candidate for the mechanism underlying our finding of increased GFS for specific oscillations and states that rely on thalamic activity. This conjecture, however, is very preliminary and future studies using simultaneous EEG and fMRI to calculate GFS and correlate it with thalamic activity during different states are necessary. Indeed, a recent study using simultaneous EEG/fMRI found thalamic involvement in the generation of microstates recorded from scalp EEG and proposed that the thalamus is important for the synchrony of oscillations among different cortical regions [31].

Our results highlight a novel aspect of REM sleep, not previously reported—namely that REM sleep exhibits high global synchrony. We propose the involvement of the thalamus in producing this synchrony and hope that this finding will spur research examining synchronous activity and its functional significance during REM sleep.

## Supplementary Material

Supplementary Figure S1: Name and location of the 27 electrodes projected onto a 2D surface used in the current study. Electrode position is at the center of the label. Supplementary Figure S2: Illustration of GFS determination in complex plain for f= 12 Hz. Circles represent single EEG derivations and the length of the grey lines corresponds to the two eigenvalues λ1 and λ2 (see Methods). Supplementary Figure S3: Subject average GFS spectra (smoothed, 9-point moving average; only for visualization purposes) of the wake EEG eyes open and eyes closed during sustained wakefulness of 40 h. Figure legend refers to the number of hours awake. Blue 0 h awake is after a baseline night of sleep while the 0 h wake in black is after a night of recovery sleep following sleep deprivation.

## Data accessibility

Our data are available for download in the supplementary section of the journal and by request from the first authors.
