# Supplementary material for: Global field synchronization reveals rapid eye movement sleep as most synchronized brain state in the human EEG
Source: R Soc Open Sci. 2016 Oct 12;3(10):160201. doi: 10.1098/rsos.160201 (PMC5098962; doi:10.1098/rsos.160201)
Supplement: Supplementary Figure S1: Name and location of the 27 electrodes projected onto a 2D surface used in the current study. Electrode position is at the center of the label. Supplementary Figure S2: Illustration of GFS determination in complex plain for f= 12 Hz. Circles represent single EEG derivations  [file rsos160201supp1.pdf]

Supplementary material

Achermann P, Rusterholz T, Dürr R, König T, Tarokh L. 2016 ***Global field synchronization reveals rapid eye movement sleep as most synchronized brain state in the human EEG.*** R. Soc. open sci. 3: 160201.
http://dx.doi.org/10.1098/rsos.160201

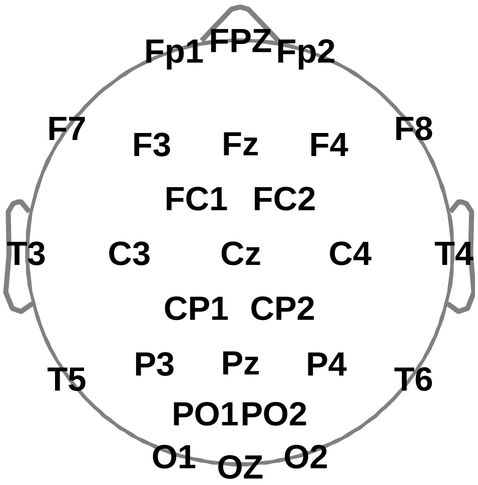

Supplementary Figure S1: Name and location of the 27 electrodes projected onto a 2D surface used in the current study. Electrode position is at the center of the label.

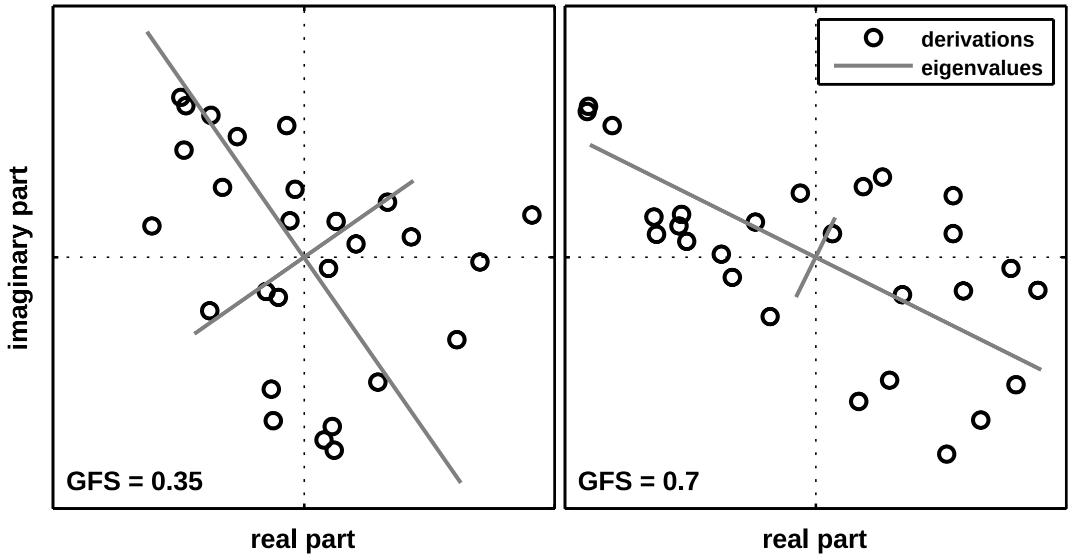

Supplementary Figure S2: Illustration of GFS determination in complex plain for f=12 Hz. Circles represent single EEG derivations and the length of the grey lines corresponds to the two eigenvalues λ_1 and λ_2 (see Methods).

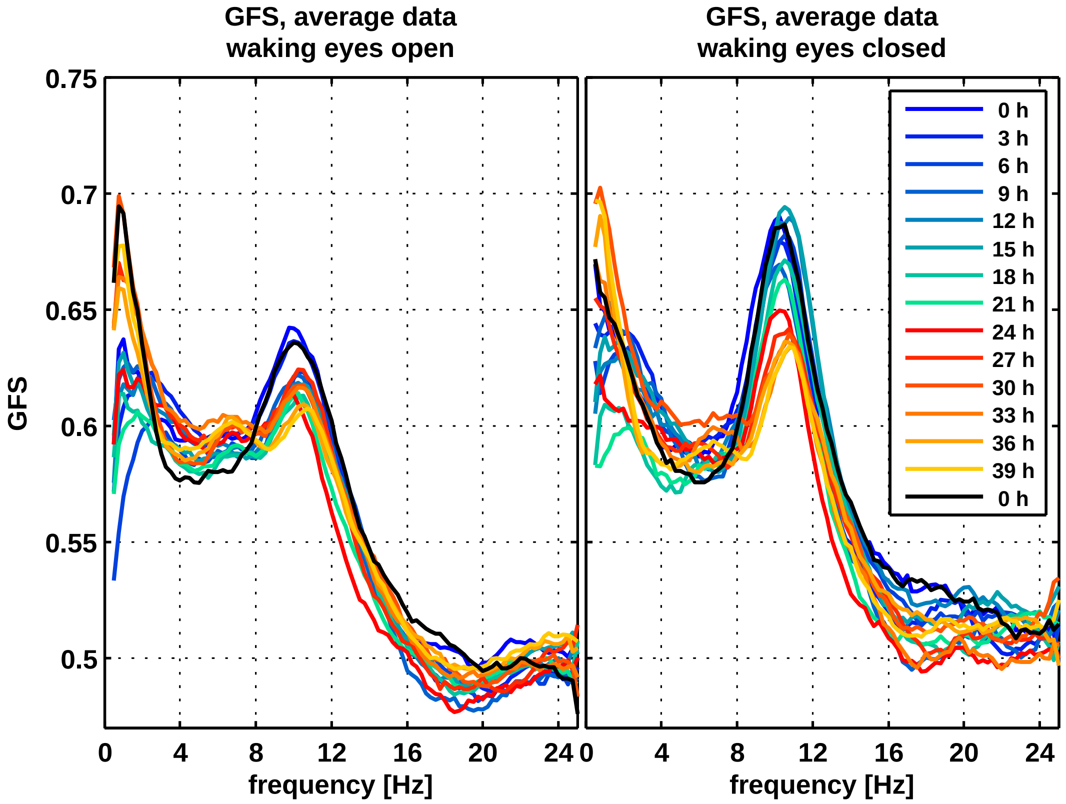

Supplementary Figure S3: Subject average GFS spectra (smoothed, 9-point moving average; only for visualization purposes) of the wake EEG eyes open and eyes closed during sustained wakefulness of 40 h. Figure legend refers to the number of hours awake. Blue 0 h awake is after a baseline night of sleep while the 0 h wake in black is after a night of recovery sleep following sleep deprivation.
